# Comparison of methods for the identification of microorganisms isolated from blood cultures

**DOI:** 10.1186/s12941-016-0158-9

**Published:** 2016-08-05

**Authors:** Aydir Cecília Marinho Monteiro, Carlos Magno Castelo Branco Fortaleza, Adriano Martison Ferreira, Ricardo de Souza Cavalcante, Alessandro Lia Mondelli, Eduardo Bagagli, Maria de Lourdes Ribeiro de Souza da Cunha

**Affiliations:** 1 Departamento de Microbiologia e Imunologia, Instituto de Biociências de Botucatu, UNESP–Univ. Estadual Paulista, Distrito de Rubião Junior, s/n, Botucatu, SP CEP: 18618-970 Brazil; 2 Departamento de Doenças Tropicais, Faculdade de Medicina de Botucatu, UNESP–Univ. Estadual Paulista, Distrito de Rubião Junior, s/n, Botucatu, SP CEP: 18618-970 Brazil; 3 Laboratório de Análises Clínicas do Hospital das Clínicas de Botucatu, Faculdade de Medicina de Botucatu, UNESP–Univ. Estadual Paulista, Distrito de Rubião Junior, s/n, Botucatu, SP CEP: 18618-970 Brazil; 4 Comissão de Controle de Infecção Relacionada à Assistência à Saúde, Hospital das Clínicas, Faculdade de Medicina de Botucatu, UNESP–Univ. Estadual Paulista, Distrito de Rubião Junior, s/n, Botucatu, SP CEP: 18618-970 Brazil; 5 Departamento de Clínica Médica, Faculdade de Medicina de Botucatu, UNESP–Univ. Estadual Paulista, Distrito de Rubião Junior, s/n, Botucatu, SP CEP: 18618-970 Brazil

**Keywords:** Blood culture, Phenotypic identification, Genotypic identification, Automated VITEK^®^ 2 system

## Abstract

**Background:**

Bloodstream infections are responsible for thousands of deaths each year. The rapid identification of the microorganisms causing these infections permits correct therapeutic management that will improve the prognosis of the patient. In an attempt to reduce the time spent on this step, microorganism identification devices have been developed, including the VITEK^®^ 2 system, which is currently used in routine clinical microbiology laboratories.

**Methods:**

This study evaluated the accuracy of the VITEK^®^ 2 system in the identification of 400 microorganisms isolated from blood cultures and compared the results to those obtained with conventional phenotypic and genotypic methods. In parallel to the phenotypic identification methods, the DNA of these microorganisms was extracted directly from the blood culture bottles for genotypic identification by the polymerase chain reaction (PCR) and DNA sequencing.

**Results:**

The automated VITEK^®^ 2 system correctly identified 94.7 % (379/400) of the isolates. The YST and GN cards resulted in 100 % correct identifications of yeasts (15/15) and Gram-negative bacilli (165/165), respectively. The GP card correctly identified 92.6 % (199/215) of Gram-positive cocci, while the ANC card was unable to correctly identify any Gram-positive bacilli (0/5).

**Conclusions:**

The performance of the VITEK^®^ 2 system was considered acceptable and statistical analysis showed that the system is a suitable option for routine clinical microbiology laboratories to identify different microorganisms.

## Background

Sepsis is a global health problem and an estimated 17 million cases of sepsis occur each year in the world [1]. The early initiation of appropriate antibiotic therapy is determinant for the prognosis and survival of patients with bloodstream infections [2]. Patients receiving antibiotic therapy that is adapted based on the susceptibility profile of the infectious agent isolated from blood cultures exhibit lower mortality than those treated initially with inadequate antibiotic therapy [3]. In addition, technological advances that permit the rapid and reliable identification of most pathogens involved in infectious diseases have long been recognized to have clinical benefits, including shorter hospital stays and lower mortality, as well as financial benefits by reducing healthcare costs [4].

The objective of this study was to evaluate the sensitivity of the VITEK^®^ 2 system, a system that automatically performs the processes required for microorganism identification and for the determination of antimicrobial susceptibility using a standard primary inoculum isolated from subcultures of positive blood cultures. Although classical identification methods are still considered the gold standard, these methods are slow, time consuming and prone to subjective interpretations. On the other hand, the VITEK^®^ 2 system reduces the time necessary for identification and permits the standardization of inter- and intra-laboratory results, the storage of results, issuing rapid epidemiological reports, and simultaneous identification and antimicrobial susceptibility testing; however, the system is poorly efficient in identifying certain species of Gram-positive cocci [42].

Studies using direct inoculation of VITEK^®^ 2 cards from blood culture bottles have been conducted in an attempt to further reduce the time of identification of microorganisms that cause bloodstream infections, but the results were acceptable only for Gram-negative bacilli and were inaccurate for Gram-positive cocci [5, 6]. For this reason, the present study used inocula of microorganisms cultured previously on solid media for 24 h.

The difference of this study was the prospective evaluation of the VITEK^®^ 2 system during the routine work of a clinical microbiology laboratory in a university hospital using 400 microorganisms isolated from blood cultures collected during the hospitalization period of patients rather than to conduct a retrospective study of samples stored for years.

## Methods

### Isolates studied

Four hundred microorganisms isolated from positive blood cultures of patients hospitalized in intensive care units of the Botucatu University Hospital between August 2012 and February 2014 were identified. The blood samples were inoculated into blood culture bottles and incubated in the BACTEC*™* 9050 apparatus.

### Identification of microorganisms in positive blood cultures

#### Automated phenotypic identification

Samples exhibiting microbial growth were submitted to Gram staining and cultured on solid media directly from the blood culture bottles. After subculture on blood and MacConkey agar, the isolates were inoculated into the following specific identification cards of the automated VITEK^®^ 2 system using the standard protocol: Gram-positive cocci (GP), Gram-positive bacilli (GN), Gram-negative bacilli (ANC), and yeasts (YST). Gram-positive cocci, Gram-positive bacilli and yeast were inoculated into the cards from colonies grown on blood agar and Gram-negative bacilli from colonies grown on MacConkey agar, all diluted in saline (0.9 % NaCl) to a 0.5 McFarland standard.

#### Phenotypic identification by conventional methods

Phenotypic identification consisted of Gram staining for the observation of morphology and specific staining, followed by a series of biochemical tests specific for each group of microorganisms. Gram-positive cocci were submitted to the catalase test for differentiation between *Staphylococcus* and *Enterococcus*. The following biochemical test battery was used for the identification of species of the genus *Staphylococcus*: coagulase, sugar fermentation (sucrose, maltose, trehalose, xylose, and mannitol), anaerobic growth on semi-solid sodium thioglycolate medium and, if necessary, ornithine and urease production and novobiocin susceptibility [7]. Isolates previously identified as Gram-positive, catalase-negative, bile esculin-positive, NaCl-positive (growth in brain heart infusion broth with 6.5 % NaCl) and pyrrolidonyl-aminopeptidase test-positive cocci were submitted to biochemical tests of fermentation of mannitol, arabinose, arginine and sorbitol, motility, and presence or absence of a pigment on sheep blood agar. Gram-negative bacilli were first tested for glucose fermentation. Glucose-fermenting bacilli were submitted to manual biochemical tests known as EPM/MILi/Citrate, an identification system based on the following tests: production of H_2_S, urease and l-tryptophan desaminase; motility; indol production; lysine decarboxylase production, and the ability to use citrate as a single carbon source. Non-glucose-fermenting Gram-negative bacilli were identified based on motility, growth at a temperature of 42 °C, and production of DNAse. Yeasts were isolated on Sabouraud agar, replated on CHROMagar, and identified based on the color, texture and shape of their colonies [8].

#### DNA extraction from the isolates

##### Extraction of bacterial DNA

Bacterial DNA was extracted directly from the blood sample of the blood culture bottle using the Illustra Kit (GE Healthcare) according to the protocol of the manufacturer, with modifications in the first centrifugation [9] and the addition of 800 µL benzyl alcohol [10]. For sample collection, the lid of the blood culture bottle was first disinfected with cotton soaked in 70 % alcohol. Next, 1.5 mL of the culture was aspirated with a sterile needle and syringe and transferred to sterile microtubes. The microtubes were centrifuged at 850*g* for 2 min and the supernatant was removed by aspiration with a micropipette and sterile tips and the supernatant stored (directly in a DNA-free microtube) at −20 °C until the time of extraction.

For DNA extraction, the sample was centrifuged at 10,000*g* for 1 min, the supernatant was discarded, and 500 µL lysozyme was added to the sediment. The mixture was vortexed, 800 µL benzyl alcohol was added, and the mixture was again shaken and centrifuged at 7000*g* for 5 min. Next, 300 µL of the supernatant was carefully removed and transferred to a new sterile microtube (which was used for extraction). Ten microliter lysozyme (10 mg/mL) was added and the microtube was left to stand at room temperature for 15 min, with vortexing every 5 min. After this period, 10 µL proteinase K (20 mg/mL) was added and the mixture was vortexed. The microtube was incubated for 15 min at 56 °C, with vortexing every 5 min. This mixture was then transferred to an extraction microcolumn and centrifuged at 11,000*g* for 1 min. The filtrate was discarded and 500 µL washing solution was added to the microcolumn. The column was again centrifuged at 11,000*g* for 3 min. The supernatant was discarded, the microcolumn was transferred to a new sterile microtube, and 200 µL Milli-Q water previously heated to 70 °C was added. The microcolumn was kept at room temperature for 1 min and centrifuged at 11,000*g* for 1 min. The columns were discarded and the filtered material was frozen until analysis by the polymerase chain reaction (PCR).

##### Extraction of yeast DNA

Yeast DNA was extracted according to the protocol proposed by McCullough et al. [11]. The isolates were seeded onto inclined Sabouraud agar and incubated for 36 h at 37 °C. A loopful of this culture was resuspended in a 2-mL tube containing 1 mL 1 M sorbitol, 125 mM EDTA, and 500 mg glass beads. The tube was shaken twice in a Precellys^®^ homogenizer for 45 s and centrifuged at 13,000*g* for 10 min. The supernatant was discarded and the sediment together with the glass beads was resuspended in 500 µL of a buffer solution containing 50 mM Tris–HCl, 50 mM EDTA and 2 % SDS and incubated for 1 h at 65 °C. After incubation, 500 µL 3 M sodium acetate was added. The mixture was homogenized by inverting the tube and kept on ice for 2 h, followed by centrifugation for 10 min at 25 °C. The supernatant was transferred to a 1.5-mL centrifugation microtube containing 1 mL ice-cold absolute ethanol, homogenized by inversion, and centrifuged for 10 min at 4 °C. The supernatant was discarded and the DNA retained on the tube wall was resuspended in 50 µL autoclaved Milli-Q water and frozen until the time of PCR.

#### Genotypic identification of the isolates

##### Polymerase chain reaction of bacteria

Gram-positive bacteria of the genus *Staphylococcus* that belonged to the group of coagulase-negative staphylococci (CoNS) were identified by internal transcribed spacer PCR (ITS-PCR) using primers targeting conserved sequences adjacent to the 16S and 23S genes: G1 (5′-GAAGTCGTAACAAGG-3′) and L1 (5′-CAAGGCATCCACCGT-3′) [12, 13].

The remaining isolates of Gram-positive cocci and Gram-negative bacilli were submitted to PCR carried out in 0.2-mL microtubes containing 15.8 µL Milli-Q water, 10 pM of the forward primer and 10 pM of the reverse primer, 100 µM triphosphate desoxyribonucleotides (GE Healthcare), 10 U Taq DNA polymerase (Biotools), 20 mM MgCl_2_-free buffer (Biotools), 0.75 mM MgCl_2_ (Biotools), and 3 µL DNA. Primers targeting conserved sequences of each species were used for DNA amplification. The temperature and time parameters and number of amplification cycles reported in the literature and described in Table 1 were used.

**Table 1:** Primers used for the identification of some bacterial species by PCR

Microorganism	Gene	Annealing temperature (°C)	Amplicon size (bp)	Reference
*Acinetobacter baumannii*	^*bla*^OXA_51-like	52	353	[44]
*Acinetobacter lwoffii*	^*bla*^OXA_154-like	52	223	[45]
*Enterobacter cloacae*	*Hsp*60 (housekeeping)	52	343	[46]
*Enterococcus faecalis*	16S chromosomal region	60	143	[47]
*Enterococcus faecium*	*sod*A (superoxide dismutase)	45	216	[21]
*Escherichia coli*	*gad*A (alpha-glutamate decarboxylase)	65	373	[48]
*Klebsiella pneumoniae*	*rpo*B (β subunit of RNA polymerase)	52	364	[49]
*Morganella morganii*	16S chromosomal region	62	809	[50]
*Proteus mirabilis*	*rsb*A (quorum sensing)	55	236	[51]
*Pseudomonas aeruginosa*	Genome fragment	55	724	[52]
*Serratia marcescens*	16S chromosomal region	52	1058	[53]
*Staphylococcus aureus*	16S chromosomal region	52	442	[54]
*Stenotrophomonas maltophilia*	*atp*D (housekeeping)	52	854	[55]
*Bacillus* spp.	*rpo*B (β subunit of RNA polymerase)	72	400	[14]
*Enterobacter aerogenes*	16SrDNA	55	280	[15]

**Fig. 1:**
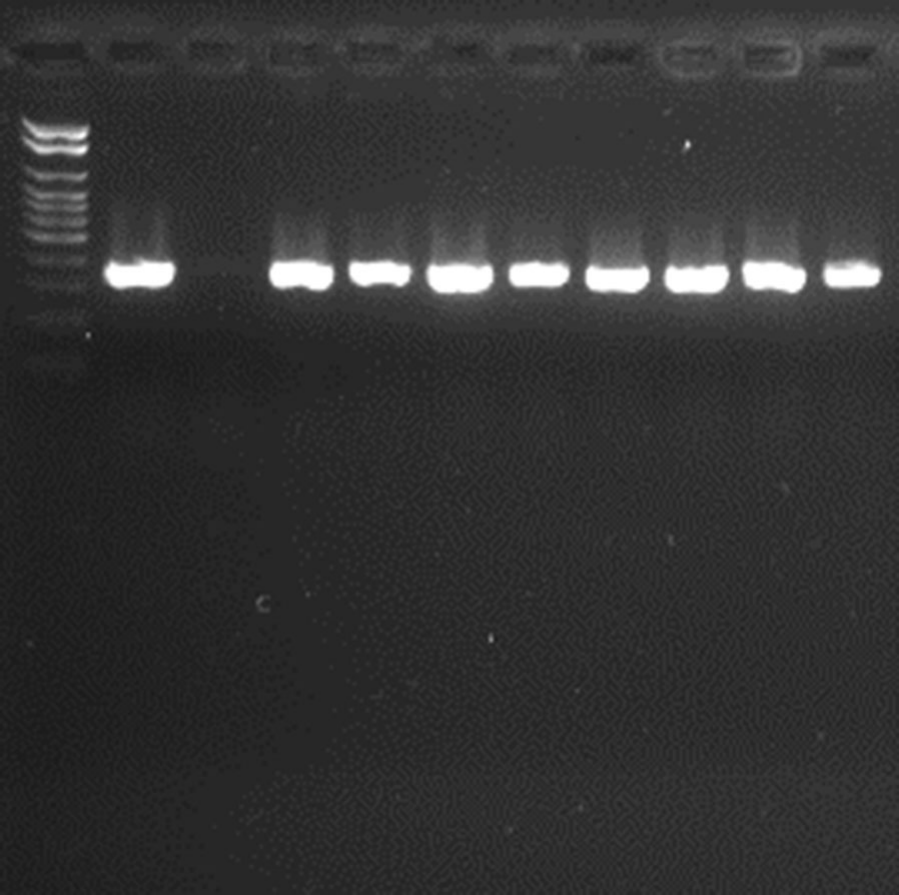
Agarose gel electrophoresis for detection of *Hsp*60 (343 bp) in *Enterobacter cloacae* (stained with SYBR^®^ Safe) showing the amplified products positive control, negative control and some samples studied. A 100-bp ladder was used as molecular size marker

**Fig. 2:**
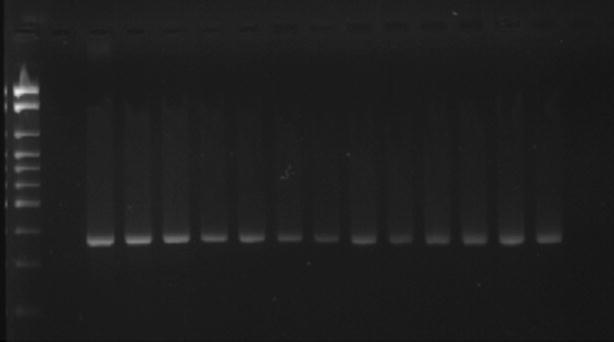
Agarose gel electrophoresis for detection of *gad*A (373 bp) in *Escherichia coli* (stained with SYBR^®^ Safe) showing the amplified products positive control, negative control and some samples studied. A 100-bp ladder was used as molecular size marker

The efficiency of the amplifications was monitored by electrophoresis on 2 % agarose gel prepared in 1× TBE buffer (89 mM Tris (pH 7.6), 89 mM boric acid, and 2 mM EDTA) and stained with SYBR^®^ Safe at 90 V for 60 min (Figs. 1, 2). The following international reference strains were used as controls: *Acinetobacter baumannii* (ATCC 19606), *Enterobacter cloacae* (ATCC 23355), *Enterococcus faecalis* (ATCC 29212), *Enterococcus faecium* (ATCC 6569), *Escherichia coli* (ATCC 10536), *Klebsiella pneumoniae* (ATCC 4352), *Morganella morganii* (ATCC 8019), *Proteus mirabilis* (ATCC 15290), *Pseudomonas aeruginosa* (ATCC 15442), *Serratia marcescens* (ATCC 14756), *Staphylococcus aureus* (ATCC 25923), and *Stenotrophomonas maltophilia* (ATCC 13637).

Figures 1 and 2 illustrate the genotypic identification of *Enterobacter cloacae* and *Escherichia coli*, respectively.

##### DNA sequencing of bacteria

The yeast and Gram-positive bacillus isolates were sequenced for identification to species level. Gram-negative bacilli identified as *Enterobacter aerogenes* by the conventional phenotypic methods and by the automated test were sequenced for species confirmation.

##### Amplification and purification of bacterial DNA for sequencing

The bacterial isolates identified as Gram-positive bacilli by Gram staining and the Gram-negative bacilli identified as *Enterobacter aerogenes* by the conventional phenotypic methods and automated test were submitted to simple PCR. The efficiency of the reactions was monitored as described in item 2.4.1. For PCR, protocols described in the literature were followed using universal primers for Gram-positive bacilli [14] and primers of enterobacteria for *Enterobacter aerogenes* isolates [15]. The temperature and time parameters and number of amplification cycles are shown in Table 1. The amplified DNA fragments were purified using the HiYield Gel/PCR DNA Fragments Extraction Kit (RBC).

##### Amplification and purification of yeast DNA

The yeast isolates were submitted to ITS-PCR as described by Kurtzman et al. [16]. These reactions were carried out in 0.2-mL microtubes containing a mixture of 6.7 µL Milli-Q water, 10 µM forward primer and 10 µM reverse primer, 12.5 µL GoTaq^®^Green Master Mix, and 3 µL DNA. The efficiency of the amplifications was monitored by electrophoresis (60 min, 80 V) on 1.5 % agarose gel prepared in 1× TBE buffer and stained with SYBR^®^ Safe. Strains previously identified at the Laboratory of Fungal Biology, Department of Microbiology and Immunology, IBB, were used as controls. The amplified fragments were purified using the Illustra™ ExoProStar™ Kit (GE Healthcare Life Sciences).

##### DNA sequencing reaction of yeast and bacteria

The sequencing reactions were carried out in a mixture containing 3.25 µL water, 1.75 µL 5× BigDye buffer (Applied Biosystems), 1 µL of each primer (5 pmol/µL), 2 µL of the PCR product, and 2 µL BigDye (Applied Biosystems). The cycle sequencing reaction was initiated at 96 °C for 1 min, followed by 40 cycles of denaturation at 96 °C for 30 s, annealing at 60 °C for 30 s and extension at 72 °C for 4 min. The sequencing reaction product was precipitated with 1 µL 125 mM EDTA, 1 µL 3 M sodium acetate, and 25 µL 100 % ethanol. After homogenization, the solution was left to stand for 15 min and then centrifuged at 3000*g* for 15 min at 4 °C. The supernatant was removed by inverting the tube and 35 µL 70 % ethanol was added. The solution was centrifuged at 1650*g* for 15 min at 4 °C. After removal of the supernatant by inversion, 10 µL HiDi formamide (Applied Biosystems) was added and the mixture was left to stand for 5 min at 95 °C and for 2 min on ice. The product was run on an 8-capillary ABI 3500 sequencer (50 cm) using POP7 polymer (Applied Biosystems).

##### Analysis of the DNA sequences of yeast and bacteria

The MEGA 5.0 (Molecular Evolutionary Genetics Analysis) and Lasergene programs were used for visualization and alignment of the DNA sequences obtained, which were compared to sequences published and stored in the GenBank database.

### Statistical analysis

Agreement between the results obtained with the different identification methods was analyzed statistically by the kappa test using the SPSS 20 program (IBM, Armonk, NY, USA). Genotypic identification was used as the gold standard. The accuracy of the conventional phenotypic identification methods and identification by the VITEK^®^ 2 system was evaluated by calculating sensitivity and specificity according to Fletcher et al. [17].

## Results

The results of identification with the automated VITEK^®^ 2 system showed overall agreement of 94.7 % with the results of the genotypic methods (Tables 2, 3). Overall agreement of 98.7 % was observed between the results obtained by phenotypic identification using conventional methods and the results of the genotypic methods.

**Table 2:** Comparison of the results of identification of blood culture isolates obtained with the automated VITEK^®^ 2 system, conventional phenotypic methods, and genotypic methods

Microorganism isolated (number)	Automated identification	Conventional methods	Genotypic identification
*Bacillus licheniformis* (N = 2)	0	NP	2
*Corynebacterium amycolatum* (N = 3)	0	NP	3
*Enterococcus faecalis* (N = 8)	7	8	8
*Staphylococcus epidermidis* (N = 81)	71	81	81
*Staphylococcus hominis* (N = 35)	32	33	35
*Staphylococcus capitis* (N = 18)	17	18	18
*Staphylococcus haemolyticus* (N = 50)	49	48	50
*Staphylococcus aureus* (N = 17)	17	17	17
*Enterobacter cloacae* (N = 13)	13	13	13
*Proteus mirabilis* (N = 5)	5	5	5
*Escherichia coli* (N = 13)	13	13	13
*Serratia marcescens* (N = 22)	22	22	22
*Acinetobacter baumannii* (N = 39)	39	39	39
*Acinetobacter lwoffii* (N = 3)	3	3	3
*Candida albicans* (N = 5)	5	5	5
*Candida glabrata* (N = 5)	5	5	5
*Candida krusei* (N = 2)	2	2	2
*Candida tropicalis* (N = 3)	3	3	3
*Enterobacter aerogenes* (N = 8)	8	8	8
*Enterococcus faecium* (N = 4)	4	4	4
*Klebsiella pneumoniae* (N = 43)	43	42	43
*Morganella morganii* (N = 3)	3	3	3
*Pseudomonas aeruginosa* (N = 15)	15	15	15
*Staphylococcus cohnii* (N = 1)	1	1	1
*Staphylococcus warneri* (N = 1)	1	1	1
*Stenotrophomonas maltophilia* (N = 1)	1	1	1

*NP* identification not performed

**Table 3:** Discrepant results between the automated VITEK^®^ 2 system and the other identification methods of blood culture isolates

No.	Automated identification	Conventional methods	Genotypic identification
4	*Staphylococcus hominis*	*Staphylococcus epidermidis*	*Staphylococcus epidermidis*
3	*Corynebacterium urealyticum*	NP	*Corynebacterium amycolatum*
2	*Corynebacterium urealyticum*	NP	*Bacillus licheniformis*
2	*Staphylococcus lentus*	*Staphylococcus epidermidis*	*Staphylococcus epidermidis*
2	*Staphylococcus haemolyticus*	*Staphylococcus hominis*	*Staphylococcus hominis*
1	*Enterococcus gallinarum*	*Enterococcus faecalis*	*Enterococcus faecalis*
1	*Staphylococcus cohnii*	*Staphylococcus capitis*	*Staphylococcus capitis*
1	*Staphylococcus haemolyticus*	*Staphylococcus haemolyticus*	*Staphylococcus epidermidis*
1	*Staphylococcus lugdunensis*	*Staphylococcus epidermidis*	*Staphylococcus epidermidis*
1	*Staphylococcus scuri*	*Staphylococcus epidermidis*	*Staphylococcus epidermidis*
1	*Staphylococcus capitis*	*Staphylococcus epidermidis*	*Staphylococcus epidermidis*
1	*Staphylococcus warneri*	*Staphylococcus haemolyticus*	*Staphylococcus haemolyticus*
1	*Staphylococcus haemolyticus*	*Staphylococcus haemolyticus*	*Staphylococcus hominis*

*NP* identification not performed

All yeast isolates (15/15) were correctly identified by the VITEK^®^ 2 system using the YST card. The same result was obtained with the GN card for the identification of Gram-negative bacilli, with 100 % correct identifications of the 165 strains isolated.

The rate of correct identification obtained with the GP card used for the identification of Gram-positive cocci was 92.6 % for the microorganisms isolated (199/215). The agreement between the results of automated identification and those obtained with the other identification methods for *Enterococcus* spp. was 91.7 % due to the incorrect identification of one *Enterococcus faecalis* strain by the VITEK^®^ 2 system. The agreement between species of the genus *Staphylococcus* was 97.5 % (198/203). The VITEK^®^ 2 system correctly identified all *Staphylococcus aureus* isolates (17/17) and incorrectly identified 15 of the 186 (9.19 %) isolates belonging to the group of CoNS. The highest rate of incorrect identification was observed for *Enterococcus faecalis* with 12.5 % (7/8), followed by *Staphylococcus epidermidis* with 12.3 % (10/81), *Staphylococcus hominis* with 8.6 % (3/35), *Staphylococcus capitis* with 5.5 % (1/18), and *Staphylococcus haemolyticus* with 2 % (1/50).

The VITEK^®^ 2 system incorrectly identified the isolates because some biochemical tests failed during the identification process performed by the device, exhibiting divergent characteristics of the species isolated. These errors are shown in Table 4.

**Table 4:** Biochemical tests that failed during the identification process by the VITEK^®^ 2 system and by the conventional phenotypic methods

No.	Genotypic identification	Conventional phenotypic method	VITEK^®^ 2 system		
	Identification	Identification	Incorrect test	Identification	Incorrect test
3	*Staphylococcus epidermidis*	*Staphylococcus epidermidis*	0	*Staphylococcus hominis*	dMNE−; TRE +
2	*Staphylococcus epidermidis*	*Staphylococcus epidermidis*	0	*Staphylococcus lentus*	PolyB−
1	*Staphylococcus epidermidis*	*Staphylococcus epidermidis*	0	*Staphylococcus cohnii*	SUC−; dTRE +
3	*Staphylococcus hominis*	*Staphylococcus hominis*	0	*Staphylococcus haemolyticus*	PyrA+; dMAN+
2	*Staphylococcus hominis*	*Staphylococcus epidermidis*	THIO+	*Staphylococcus hominis*	0
2	*Staphylococcus haemolyticus*	*Staphylococcus warneri*	URE+	*Staphylococcus haemolyticus*	0
1	*Enterococcus faecalis*	*Enterococcus faecalis*	0	*Enterococcus gallinarum*	dSOR−; dRAF+
1	*Staphylococcus capitis*	*Staphylococcus capitis*	0	*Staphylococcus cohnii*	βGAL+; βGUR+; SUC−; dTRE+
1	*Staphylococcus epidermidis*	*Staphylococcus haemolyticus*	URE−	*Staphylococcus haemolyticus*	PyrA+; URE−, PolyB−; TRE+; dMNE−
1	*Staphylococcus epidermidis*	*Staphylococcus epidermidis*	0	*Staphylococcus lugdunensis*	PyrA+; dTRE+
1	*Staphylococcus epidermidis*	*Staphylococcus epidermidis*	0	*Staphylococcus scuri*	PolyB−; βGUR+; NAG (+); dMAN+; dTRE+
1	*Staphylococcus epidermidis*	*Staphylococcus epidermidis*	0	*Staphylococcus capitis*	SUC−; dMAL−; PolyB−; URE−
1	*Staphylococcus haemolyticus*	*Staphylococcus haemolyticus*	0	*Staphylococcus warneri*	0
1	*Klebsiella pneumoniae*	*Klebsiella oxytoca*	Indol+	*Klebsiella pneumoniae*	0

*dMNE* d-mannose, *dTRE* d-trehalose, *PolyB* polymyxin B, *SUC* sucrose, *PyrA* l-pyrrolidonylarylamidase, *dMAN* d-mannitol, *dSOR* d-sorbitol, *dRAF* d-raffinose, *URE* urea, *βGAL* beta-galactosidase, *THIO* thioglycolatebroth, *βGUR* beta-glucuronidase, *NAG* *N*-acetylglucosamine, 0 no errors in the tests

Fewer errors occurred when the conventional phenotypic methods were used for identification compared to the automated VITEK^®^ 2 system. Among Gram-positive cocci, the conventional phenotypic methods correctly identified 211/215 (98.1 %) isolates and the few errors observed mainly occurred in the identification of CoNS species, showing divergent results for a given species. The conventional phenotypic methods correctly identified all yeasts (15/15) and 164/165 (99.4 %) Gram-negative bacilli, with the errors described in Table 4.

One hundred percent discrepant results were obtained for the identification of five isolates of Gram-positive bacilli by the VITEK^®^ 2 system (ANC card) and the genotypic identification methods.

Statistical analysis of the identification results revealed a kappa value of 0.945 (p < 0.001), indicating almost perfect agreement according to the criteria of Landis and Koch [18] (Table 5).

**Table 5:** Kappa value according to Landis and Koch

Kappa value	Level of agreement
<0.00	No agreement
0.00–0.20	Slight
0.21–0.40	Fair
0.41–0.60	Moderate
0.61–0.80	Substantial
0.81–1.00	Almost perfect

Agreement between the conventional phenotypic methods and genotypic identification was higher than that between the genotypic method and automated identification by the VITEK^®^ 2 system (Table 6).

**Table 6:** Kappa values of agreement between automated identification by the VITEK^®^2 system, the conventional phenotypic methods, and genotypic identification

Group	Number of isolates	Kappa	
		Conventional method × genotypic method	Automated method × genotypic method
Gram-positive cocci	255	0.969	0.904
CoNS	186	0.969	0.886
Gram-negative bacilli	165	0.993	1.000
Gram-positive bacilli	5	NP	0
Yeasts	15	1.000	1.000
Total	400	0.958	0.945

*CoNS* coagulase-negative staphylococci, *NP* not performed

Comparison of the sensitivity of the conventional phenotypic methods and the VITEK^®^ 2 system showed a better performance of the former (Table 7).

**Table 7:** Sensitivity and specificity of the conventional phenotypic methods and of the automated VITEK^®^ 2 system in the identification of microorganisms

Microorganism	VITEK^®^ 2 system		Phenotypic methods	
	Sensitivity (%)	Specificity (%)	Sensitivity (%)	Specificity (%)
*Enterococcus faecalis*	87.5	100	100	100
*Staphylococcus capitis*	94.4	99.5	100	100
*Staphylococcus cohnii*	100	99.0	100	100
*Staphylococcus epidermidis*	87.6	100	100	98.5
*Staphylococcus haemolyticus*	98.0	96.5	96.0	100
*Staphylococcus hominis*	91.4	98.3	94.2	100
*Staphylococcus warneri*	100	99.5	100	99.0

## Discussion

The need for the rapid and efficient identification of microorganisms isolated from blood cultures has encouraged studies that investigated automated identification systems to reduce the time of identification. Several of these studies have used direct inoculation from blood culture bottles, but the results were not as efficient as those obtained in studies using standard inocula from subcultures of microorganisms grown for 24 h on solid media. The poor performance of the VITEK^®^ 2 system for the identification of microorganisms using direct inoculation from blood culture bottles is probably due to the small number of cells or to contamination with other microorganisms that impair the correct identification of the causative agent of infection [19–22]. The VITEK system has been investigated for more than two decades and improvement of the ID-GPC (Gram-positive cocci) and ID-GNB (Gram-negative bacilli) identification cards to the GP (Gram-positive cocci) and GN (Gram-negative bacilli) cards has made the system more efficient. Wallet et al. [23] compared the old and new identification cards and found that the GP and GN cards correctly identified 235/249 (94.4 %) Gram-positive cocci and 321/331 (97 %) Gram-negative bacilli, while the ID-GPC and ID-GNB correctly identified 218/249 (87.5 %) and 295/331 (89.1 %) isolates, respectively.

The present study was conducted over a period of 18 months and evaluated the accuracy of the VITEK^®^ 2 system in identifying 400 microorganisms (Gram-positive cocci, Gram-positive bacilli, Gram-negative bacilli, and yeasts) isolated from blood cultures and inoculated by the standard method onto GP, GN, YST (yeast) and ANC (Gram-positive bacilli) cards. The results were compared to genotypic identification (gold standard) and 94.7 % agreement was observed. Similar rates have been reported by De Cueto et al. [24] (95.0 %, 95/100) and by Nakasone et al. [25] (95.8 %, 454/474) who also used standard inocula. Studies using direct inoculation from blood cultures bottles obtained lower agreement of 91.4 % [5] and 81.0 % [6].

All 15 yeasts isolated during the study period were correctly identified by the VITEK^®^ 2 system. Correct identification of all yeast isolates (56/56) has also been observed by Nakasone et al. [25]. Studies involving a larger number of strains and species found lower agreement, 92.1 % (222/241) reported by Graf et al. [26] and 78.9 % (277/351) by Won et al. [27].

The VITEK^®^ 2 system has shown satisfactory identification rates of Gram-negative bacilli for decades, as also observed in this study in which 100 % correct identifications of the isolates were obtained. Funke et al. [28] and Ling et al. [29] analyzed 845 and 281 isolates, with correct identification rates of 84.7 % (716/845) and 95 % (267/281), respectively. Nakasone et al. [25], Gherardi et al. [5] and Prod’hom et al. [6] analyzed 181, 91 and 95 Gram-negative bacilli and obtained correct identifications of 96.7 % (175/181), 100 % (91/91) and 98.8 % (92/95), respectively, with the VITEK^®^ 2 system. Studies using direct inoculation from blood culture bottles reported lower rates of correct identification of 82 [30] and 93 % [31]. De Cueto et al. [24] compared direct inoculation from blood cultures with inoculation from subcultures in 50 isolates. The result was 100 % correct identifications for the standard method and 62 % (31/5) correct identifications for direct inoculation. Similar results have been reported by Kerremans et al. [32] who analyzed 161 isolates; 90 % (145/161) of the isolates were correctly identified by subculture and 80 % (129/161) by direct inoculation from blood cultures.

The identification of Gram-positive cocci by the automated VITEK^®^ 2 system showed 92.6 % agreement with genotypic identification, which is compatible with the rate reported by Ligozzi et al. [33] who obtained 91.5 % (351/381) correct identifications. De Cueto et al. [24] obtained 100 % (50/50) correct identifications of the isolates. Funke and Funke-Kissling [34], Nakasone et al. [25] and Chatzigeorgiou et al. [35] reported higher rates of correct identification than those obtained in this study of 94.5 % (344/364), 96.1 % (226/235) and 97.9 % (144/147), respectively. Gherardi et al. [5] and Prod’hom et al. [6] obtained correct identifications of 75 % (36/48) and 74 % (133/180), while Lupetti et al. [36] found a rate of 89 % (49/55). These rates are lower than those observed in this study and are outside the acceptable parameter of 90 % correct identifications; however, these studies used direct inoculation from blood culture bottles.

With respect to genera of Gram-positive cocci, a difference in the efficiency of the VITEK^®^ 2 system was observed. Agreement was 91.7 % (11/12) for the genus *Enterococcus*, similar to the rates reported by Nakasone et al. [25] and Jin et al. [37]. Lower efficiencies of 77.8, 83.1, 87.5, 72 and 77.8 % correct identifications of strains of this genus have been observed by Ligozzi et al. [33], d’Azevedo et al. [38], Moore et al. [39] and Paim et al. [42], respectively. In the case of *Staphylococcus*, a higher rate of incorrect identifications was observed for CoNS isolates (91.9 %), in agreement with Ligozzi et al. [33] (86 %), Funke and Funke-Kissling [34] (93.7 %), Kim et al. [40] (87.5 %), Delmas et al. [41] (78.8 %), and Paim et al. [42] (72.9 %). These unsatisfactory results are due to the fact that automated identification systems are unable to perform a fully reliable differentiation between different CoNS species because of the variable expression of phenotypic characteristics in these microorganisms [43]. The slow metabolism of certain species leads to ambiguous results in their identification, a fact observed by Ligozzi et al. [33]. All *Staphylococcus aureus* isolates were correctly identified (17/17), as also reported in the studies of Delmas et al. [41] (6/6), Chatzigeorgiou et al. [35] (52/52), Paim et al. [42] (11/11), and Funke and Funke-Kissling [34] (45/45). Ligozzi et al. [33], who evaluated a larger number of isolates, found 99 % agreement (99/100). These rates of correct identification demonstrate a satisfactory performance of the VITEK^®^ 2 system for the identification of *Staphylococcus aureus*.

The failure of the ANC card to identify Gram-positive bacilli can be explained by the variability in the genera and species of these microorganisms and the consequent difficulty in developing cards that contain variable biochemical tests necessary for correct identification. These microorganisms were not identified by conventional phenotypic methods since this identification is infeasible in routine clinical microbiology because it requires numerous expensive and time-consuming biochemical tests.

The better performance of the conventional methods for CoNS identification was responsible for the higher sensitivity of these methods compared to the VITEK^®^ 2 system. This finding can be explained by the fact that the conventional methods used consisted of specific tests for each CoNS species and by the incubation period of 72 h, which is necessary for this correct identification since some species have a slower metabolism on some substrates.

## Conclusions

The kappa values indicate reliability of the results obtained with the VITEK^®^ 2 system. Analysis of specificity showed a performance higher than 90 % as required for commercial systems in clinical microbiology, demonstrating that this system is suitable for the identification of microorganisms isolated in routine clinical microbiology laboratories.
